# Identification of candidate mimicry proteins involved in parasite-driven phenotypic changes

**DOI:** 10.1186/s13071-015-0834-1

**Published:** 2015-04-15

**Authors:** Francois Olivier Hebert, Luke Phelps, Irene Samonte, Mahesh Panchal, Stephan Grambauer, Iain Barber, Martin Kalbe, Christian R Landry, Nadia Aubin-Horth

**Affiliations:** Institut de Biologie Intégrative et des Systèmes (IBIS), Département de Biologie, Université Laval, Pavillon Charles-Eugènes-Marchand, Québec, G1V 0A6 Canada; Department of Evolutionary Ecology, Max Planck Institute for Evolutionary Biology, August-Thienemann-Str 2, 24306 Ploen, Germany; Department of Biology, Adrian Building, Leicester University, University Road, Leicester, LE1 7RH UK

**Keywords:** Parasites, *Schistocephalus solidus*, Cestodes, Ecological genomics, Genomics/proteomics, Molecular mimicry, Host-parasite interactions, Wnt, RT-PCR

## Abstract

**Background:**

Endoparasites with complex life cycles are faced with several biological challenges, as they need to occupy various ecological niches throughout their development. Host phenotypes that increase the parasite’s transmission rate to the next host have been extensively described, but few mechanistic explanations have been proposed to describe their proximate causes. In this study we explore the possibility that host phenotypic changes are triggered by the production of mimicry proteins from the parasite by using an ecological model system consisting of the infection of the threespine stickleback (*Gasterosteus aculeatus*) by the cestode *Schistocephalus solidus*.

**Method:**

Using RNA-seq data, we assembled 9,093 protein-coding genes from which ORFs were predicted to generate a reference proteome. Based on a previously published method, we built two complementary analysis pipelines to i) establish a general classification of protein similarity among various species (pipeline A) and ii) identify candidate mimicry proteins showing specific host-parasite similarities (pipeline B), a key feature underlying the possibility of molecular mimicry.

**Results:**

Ninety-four tapeworm proteins showed high local sequence homology with stickleback proteins. Four of these candidates correspond to secreted or membrane proteins that could be produced by the parasite and eventually be released in or be in contact with the host to modulate physiological pathways involved in various phenotypes (e.g. behaviors). One of these candidates belongs to the Wnt family, a large group of signaling molecules involved in cell-to-cell interactions and various developmental pathways. The three other candidates are involved in ion transport and post-translational protein modifications. We further confirmed that these four candidates are expressed in three different developmental stages of the cestode by RT-PCR, including the stages found in the host.

**Conclusion:**

In this study, we identified mimicry candidate peptides from a behavior-altering cestode showing specific sequence similarity with host proteins. Despite their potential role in modulating host pathways that could lead to parasite-induced phenotypic changes and despite our confirmation that they are expressed in the developmental stage corresponding to the altered host behavior, further investigations will be needed to confirm their mechanistic role in the molecular cross-talk taking place between *S. solidus* and the threespine stickleback.

**Electronic supplementary material:**

The online version of this article (doi:10.1186/s13071-015-0834-1) contains supplementary material, which is available to authorized users.

## Background

Interspecific interactions among trophic levels can act as powerful drivers of biodiversity. Among the many possible ecological interactions that modulate energetic transfers through natural ecosystems, host-parasite interactions come up as the most frequent and widespread components of food webs [1]. Several million years of evolution led to the development of extremely diverse parasitic lifestyles, ranging from broad generalists having the potential to infect various species acting as their single host, to highly specialized species seeking the shelter of several specific intermediate hosts [2,3]. Endoparasites with complex life cycles are faced with several biological challenges, as they need to occupy various ecological niches throughout their development. This strategy requires them to keep their current host alive and ultimately find their way into a final host that is indispensable for reproduction [4]. Intermediate hosts involved in these complex life cycles can exhibit drastic parasite-driven phenotypic alterations that enhance the parasite’s transmission rate, by making them more vulnerable to predation by the next host for instance [5]. As an example, rats infected with *Toxoplasma gondii* lose their fear of their predator as they become attracted by the smell of feline urine, thus increasing the parasite’s chances of transmission to its mammalian definitive host [6,7]. One way of understanding such complex ecological interactions consists of characterizing the molecular cross-talk taking place between the parasite and its hosts [8].

Evidence from molecular analyses looking at the interaction between *T. gondii* and its murine host suggests that the behavioral change observed in infected rats is partly achieved through the expression of a tyrosine hydroxylase enzyme encoded in the parasite’s genome. Interestingly, this protein is homologous to the one found in the host and directly alters dopamine levels in the rodent’s brain [9]. Such empirical evidence suggests that one molecular mechanism that can be proposed to explain some of these behavior alterations by parasites involves the use of structural similarities between molecules, a phenomenon coined “molecular mimicry”. The term molecular mimicry was first proposed by R. Damian [10] to describe antigen sharing between a parasite and its host. Consistent with this original concept, we use it here to define any molecular structure from the parasite that is similar to a corresponding host molecular structure and can thus potentially give an advantage to the parasite because of their shared similarity [11]. Some parasites use molecular mimicry to subvert host defenses as they express surface molecules similar to their host’s antigens, therefore acting as a convenient camouflage [12]. Intracellular parasites can also produce mimicry molecules that interact with specific host proteins allowing them to maximize their cytoadherence (*Trypanosoma cruzi*: [13,14], *Plasmodium falciparum*: [15,16]). Additionally, molecular mimicry can be a very powerful manipulative tool allowing the parasite to modify or suppress specific pathways in the host (e.g. hormonal messages, see [17-20]). When this strategy is pushed to the extreme, it can lead to serious behavioral changes. For instance, studies suggest that endoparasites like nematomorph hairworms could induce a water-seeking behavior in their orthopteran host (e.g. crickets, grasshoppers) by expressing mimicry signaling molecules likely to be involved in this unusual suicidal behavior [21,22]. This is one of the rare cases for which empirical evidence has been brought forward to explain the proximate causes of these behavioral changes [23]. Even though the consequences of being infected by “manipulative parasites” have been extensively described, the upstream causes of these phenotypic changes have not received enough attention yet to fully explain why and how infected individuals behave differently [8].

There are many examples of host-parasite interactions involving drastic changes in host phenotype. We chose to study the model system consisting of the infection in the threespine stickleback (*Gasterosteus aculeatus*) by the cestode *Schistocephalus solidus* as it allows us to test several possibilities with regards to molecular mechanisms [24]. *Schistocephalus solidus* is a trophically transmitted tapeworm with a complex life cycle involving two intermediate hosts. The definitive host is generally a piscivorous bird, but it can be any warm-blooded vertebrate [24]. Adult worms use the bird gut to complete the final stages of sexual maturation (i.e. egg production). Eggs released into the water through the bird’s feces hatch to produce ciliated coracidia that will be trophically transmitted to any cyclopoid copepod (first intermediate invertebrate host). During the growth phase of the parasite, i.e. before becoming infective, copepods show an increased anti-predator response, which prevents potential premature transfer to the next host [25]. When larvae reach the infective stage (procercoid), copepods exhibit a reduced anti-predator behavior, leading to an increased transmission rate to the next host [26,27]. Infective procercoids will thus eventually find their way into the second obligatory intermediate host, the threespine stickleback (the only species they can successfully infect as second intermediate host, reviewed in [28]). Sticklebacks become infected when they feed on parasitized copepods, and after a few hours in the fish digestive track, procercoids will penetrate the wall of the intestine and migrate into the body cavity of the fish [29]. From there, they will transform into small plerocercoid worms that will grow to very large sizes, sometimes reaching the same mass as their host [30]. Phenotypic effects of parasitism include global physiological changes (e.g. altered reproductive potential, reviewed in [31] and altered immune response, see [32,33]), change in prey choice [34] and a partial loss of competitive ability [35]. The time when the plerocercoids reach the developmental stage at which they could reproduce in their final bird host coincides with drastic changes in the stickleback’s behavior resulting in increased predation rates by the definitive host [36,37]. Behavioral changes in the stickleback include decreased shoaling behavior [38], loss of anti-predator behavior and increased risk-taking behavior [39-42]. Although *S. solidus* infects the body cavity of its host and not the central nervous system, differences in metabolism and concentrations of neuromodulators (i.e. serotonin, epinephrine) are observed between infected and uninfected wild-caught sticklebacks [43].

There is extensive data on the physiological and behavioral impact of *S. solidus* on the stickleback [24,44], but to date, very few molecular mechanisms have been proposed to explain the proximate causes of these changes. Particularly, there is currently no empirical evidence pointing towards the existence or the type of signal that could be released by the worm to affect multiple host phenotypes (whether it is directly or indirectly triggered). Consequently, we investigated the possibility that *S. solidus* could take advantage of molecular mimicry to change its host phenotype (e.g. behavior, immunity, reproduction) using an iterative sequence similarity comparison approach. To do so, we first built a reference transcriptome for *S. solidus* from which we predicted protein sequences. We adapted a previously published method [11] to study molecular mimicry among these predicted tapeworm proteins by building two different pipelines aiming at i) establishing a general classification of protein similarity among various parasite, host and non-host species (pipeline A) and ii) identifying candidate mimicry proteins showing specific host-parasite similarities between *S. solidus* and the stickleback (pipeline B). If *S. solidus* relies on the use of molecular mimicry to change some of its host’s phenotypes, being an extracellular parasite, it will have to express at its cell surface or secrete one or multiple types of effector molecules at one point during the infection. We can thus predict that the most plausible protein candidates involved in the development of a molecular signal triggered by the parasite and effective over a distance, either directly or indirectly (i.e. through physiological cascade that ultimately affects the host’s central nervous system), will likely be secreted proteins. We confirmed that the candidate genes, selected by their signal peptide (i.e. secretory signal) and high similarity between *S. solidus* and its stickleback host, were expressed in three different developmental stages of the parasite, i.e. non-infective (no host behavioral change), infective (significant host behavioral change) and post-reproduction adult (after egg production in the final host). This first candidate validation serves as a stepping-stone towards a fully functional characterization of the molecular interaction occurring between *S. solidus* and its second intermediate host.

## Methods

### *Schistocephalus solidus* transcriptome assembly

Worms used to produce the transcriptome were collected in two different populations, one in Norway and one in Germany. RNA was extracted using Macherey-Nagel’s NucleoSpin® commercial kit (Düren, Germany) according to the manufacturer’s protocol. Two different 454 libraries were produced (GS-FLX platform), each containing eight pooled worms collected at three different time points: i) five weeks post-infection (four worms), ii) seven weeks post-infection (two worms) and iii) nine weeks post-infection (two worms) [EMBL:ERS551497, EMBL:ERS551498]. Worms used to produce these 454 libraries covered three developmental stages that can be found within a fish host, i.e. non-infective (parasite mass < 50 mg, no change in host behavior), infective (parasite mass > 50 mg, significant changes in host behavior) and the transition stage from non-infective to infective.

Raw reads were first cleaned using NGS backbone [45] based on quality and length thresholds (PHRED score ≥ 20, read length ≥ 100 nucleotides). Cleaned reads were subsequently assembled *de novo* using a combination of MIRA 4.0 [46] and RAY 2.3.0 [47]. The MIRA algorithm is an overlap-layout-consensus method, which uses trace signals and additional sequence information whereas the RAY algorithm is a k-mer-based method relying on a de Bruijn graph. To run MIRA, we used the default parameters to perform transcript assembly (job = est, denovo, accurate). Contigs tagged by MIRA as “repetitive”, i.e. chimeras generated with highly repetitive reads [48], were discarded after protein ID validation with *blastx* 2.2.29 [49], using different protein databases (swissprot, nr, ftp.ncbi.nlm.nih.gov, accessed on 12/2014). For the second assembly, we took advantage of RAY’s “additive Multiple-k” method [50] by pooling contigs obtained with different k-mer values (k = 41, 43, 45, 47, 49). We then used an incremental clustering implemented in the program CD-HIT-EST [51,52] to remove redundancy and to generate the longest and most accurate contigs possible (see Additional file 1 for details and threshold values).

After generating two independent “cleaned datasets”, contigs from both assemblies were locally aligned (*blastn*, [49]) against a raw version of the *Schistocephalus solidus* genome (50 Helminth Genomes Initiative, ftp.sanger.ac.uk/pub/pathogens/HGI/). Contigs with either no hit found in the genome or showing low quality blast results were filtered out (e-value threshold = 1e-15). This procedure was carried out to eliminate potential cDNA contamination from the host fish from which the parasite worm was extracted, as well as chimeras and false gene sequences. The two datasets were then compared to each other using CD-HIT-EST-2D [53] to identify shared sequences. We applied the same similarity and length coverage thresholds as previously used with CD-HIT-EST. Using custom-made Python scripts (https://github.com/fohebert/Scripts.git), we discarded short redundant sequences identified by CD-HIT-EST-2D (thus eliminating redundancy in the reference transcriptome) and excessively long representative sequences (>10,000 nucleotides) more likely to regroup repeated sequences and chimeras, i.e. multiple different contigs aligning on one very long contig [53,54]. Remaining contigs formed the final dataset.

Using the EMBOSS function *getorf* [55], we obtained all possible ORFs and predicted amino acid sequences (forward and reverse) for every contig retained in the final dataset. For each contig, the longest ORF was selected. We finally used *blastx* to retrieve the identity of these sequences according to local databases created with several parasite proteomes (Table 1). This highly filtered dataset specific to *Schistocephalus solidus* was used as a reference proteome for further analyses.

**Table 1:** **Species used as control, host and parasite references for protein identification**

**Species**	**Common name**	**Phylum (class)**	**Source**
**Host**			
*Gasterosteus aculeatus*	Threespine stickleback	Chordata (Actinopterygii)	Ensembl
**Non-host control fishes**			
*Xiphophorus maculatus*	Platyfish	Chordata (Actinopterygii)	Ensembl
*Oryzias latipes*	Medaka	Chordata (Actinopterygii)	Ensembl
*Lepisosteus oculatus*	Spotted gar	Chordata (Actinopterygii)	Ensembl
*Takifugu rubripes*	Japanese puffer	Chordata (Actinopterygii)	Ensembl
**Parasites**			
*Brugia malayi*	Filarial nematode	Nematoda (Onchocercidae)	Uniprot
*Cryptosporidium parvum*	---	Apicomplexa (Conoidasida)	CryptoDB
*Echinococcus granulosus*	Dog tapeworm	Plathelminthes (Cestoda)	Sanger
*Echinococcus multilocularis*	Fox tapeworm	Plathelminthes (Cestoda)	Sanger
*Giardia lamblia*	---	Metamonada (Eopharyngia)	GiardiaDB
*Hymenolepis microstoma*	Rodent tapeworm	Plathelminthes (Cestoda)	Sanger
*Leishmania major*	---	Euglenoza (Kinetoplastidia)	TritrypDB
*Plasmodium falciparum*	Malaria	Apicomplexa (Aconoidasida)	PlasmoDB
*Schistosoma mansoni*	Blood fluke	Plathelminthes (Trematoda)	Sanger
*Trypanosoma cruzi*	Chagas	Euglenoza (Kinetoplastidia)	TritrypDB
*Taenia solium*	Pork tapeworm	Plathelminthes (Cestoda)	GeneDB
*Trichomonas vaginalis*	---	Metamonada (Parabasalia)	Uniprot
**Controls**			
*Anopheles gambiae*	African malaria mosquito	Arthropoda (Culicidae)	Vectorbase
*Arabidopsis thaliana*	Mouse-ear cress, thale cress	Magnoliophyta (Magnoliopsida)	EBI
*Caenorhabditis elegans*	“Roundworm”	Nematoda (Chromadorea)	Wormbase
*Ciona intestinalis*	Vase tunicate, Sea squirt	Chordata (Ascidiacea)	JGI
*Schizosaccharomyces pombe*	Fission yeast	Ascomycota (Schizosaccharomycetes)	EBI
*Capitella teleta*	---	Annelida (Polychaeta)	Ensembl
*Trichoplax adaherens*	---	Placozoa (Tricoplacia)	Uniprot

#### Control and host proteome files

Protein sequences for protein-coding genes from completely sequenced genomes were downloaded from ftp.ebi.ac.uk (*Arabidopsis thaliana*, *Schizosaccharomyces pombe*), ftp.wormbase.org (*Caenorhabditis elegans*), http://uniprot.org (*Brugia malayi*, *Trichonomas vaginalis*, *Trichoplax adhaerens*), ftp.vector-base.org (*Anopheles gambiae*), http://cryptodb.org (*Cryptosporidium parvum*), ftp.sanger.ac.uk (*Echinococcus granulosus*, *Echinococcus multilocularis*, *Hymenolepis microstoma, Schistosoma mansoni*), http://giardiadb.org (*Giardia lamblia*), http://tritrypdb.org (*Leishmania major*, *Trypanosoma cruzi*), http://plasmodb.org (*Plasmodium falciparum* 3D7), http://broadinstitute.org (*Gasterosteus aculeatus*), ftp://ftp.ensemblgenomes.org (*Capitella teleta, Xiphophorus maculatus, Oryzias latipes, Lepisosteus oculatus, Takifugu rubripes*) and http://genedb.org (*Taenia solium*). These proteomes were used either as controls for conserved sequences (various non-parasitic species), non-host fish controls or parasite-specific sequences (parasitic species used throughout the assembly process), while the genome of *G. aculeatus* (ftp://ftp.ensemblgenomes.org) was used as the host genome (Table 1).

#### Pipeline A - general parasitic protein similarity analysis

In a first exploratory phase, the proteomes of *S. solidus* as well as six other worms were compared to the stickleback proteome using *blastp*. This procedure was carried out to verify if the stickleback proteome shares a higher similarity with its parasite proteome than various other parasitic and non-parasitic free-living worms that do not have a specific co-evolutionary background with the host. Among these six proteomes, four are from parasite species closely related to *S. solidus* (phylum *Cestoda*: *Echinococcus granulosus*, dog tapeworm; *Echinococcus multilocularis,* fox tapeworm; *Hymenolepis microstoma*, rodent tapeworm; *Taenia solium*, pork tapeworm). The two other proteomes belong to non-parasitic free-living worms, i.e. *Caenorhabditis elegans* (phylum *Nematoda*) and *Capitella teleta* (phylum *Annelida*). *C. teleta* was chosen mainly because it is a non-parasitic marine polychaete that belongs to a phylogenetic group sharing a common ancestor with cestodes that is more recent than the common ancestor between cestodes and nematodes [56]. It thus acts as a solid non-parasitic control that is closely related to *S. solidus*.

**Figure 1:**
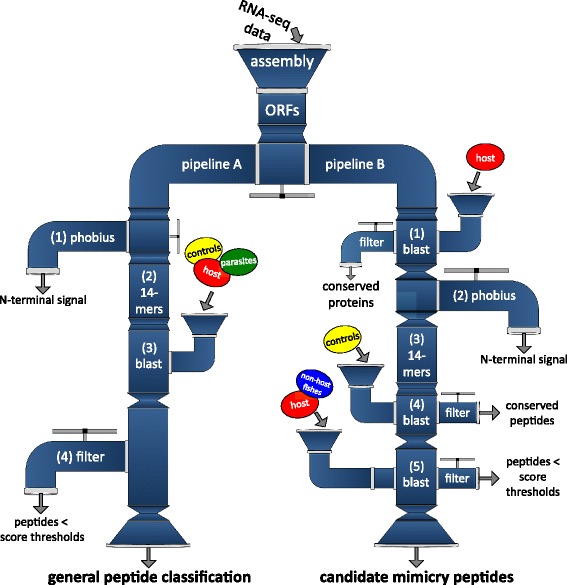
Mimicry protein identification performed *in silico* on the proteome of *Schistocephalus solidus*. Raw reads from RNA-seq data were assembled (*de novo*) into transcript sequences that were subsequently translated into ORFs. The longest ORF for each transcript was then selected and used as the reference protein sequence. The complete set of longest ORFs was used as the “predicted proteome” for downstream analyses into pipelines A & B. Pipeline A resulted in a general classification of protein similarity among diverse phylogenetic groups, while identifying few significant candidates. Pipeline B however, being more specific, resulted in the identification of significant candidate mimicry peptides involved in central nervous system functions and post-translational modification of proteins.

Several virulence factors previously identified in pathogens show strong sequence and structural homology to host proteins [57]. However, other pathogenic effectors show no apparent sequence similarity to any host protein, but display mimicry for short fragments of the protein only (e.g. tyrosine phosphatases in *Yersina* spp, [58,59] and *Salmonella* spp., [60,61]). Additional sequence similarity analyses based on fragmented proteins between *S. solidus* and its host were thus required to detect such cases of more cryptic molecular mimicry. This task was performed through pipelines A and B. First, pipeline A was used with fragmented *S. solidus* proteins and *blastp* searches against the proteomes of i) control, ii) parasite and iii) host species (Table 1) to test for general protein similarity between the parasite and various phyla (Figure 1, pipeline A). This procedure enabled us to label fragmented proteins according to their species specificity, i.e. with which species they share a certain degree of similarity (conserved vs. species-specific proteins/peptides). The following steps describing the details of pipeline A are inspired from the method presented in [11] to address the issue of sequence similarity at the protein fragments level, but was adapted to our study system. Most of the pipeline parameters found in [11] are re-used here, with minor changes in the peptide selection process, as we wanted to generate a general classification of protein similarity first. To do so, we used additional proteomes and we did not discard any peptide, but labeled them instead, according to their blast score. More specifically, a hidden Markov Model (trained on all swissprot entries) implemented in the program PHOBIUS [62] was used to predict N-terminus signals in *S. solidus* proteins. We trimmed out these short sequences using custom-made Python scripts (https://github.com/fohebert/Scripts.git) based on PHOBIUS output to eliminate potential false positives, as N-terminus signals can be shared among various types of proteins [62]. Proteins were then cleaved into short overlapping fragments of 14 residues (14-mers) with an incremental sliding window of one. General protein similarity was obtained by performing sequential *blastp* searches of *S. solidus* 14-mers against control, parasite and host species. Queries with ungapped *blastp* identities above pre-defined thresholds (Additional file 2, as defined in [11]) were considered highly similar to their hit sequence. The choice of k-mer length was made based on the method developed by Ludin et al. [11], as similarity thresholds for this particular length were tested and empirically validated (see Additional file 2). Peptides of 14 residues also represent a fair compromise between specificity and sensitivity, since short sequences are prone to align everywhere on the proteome (thus less specific), whereas long peptides can potentially return a high rate of false negatives (being too stringent). Results were then used to build a preliminary classification of protein specificity and to get an overview of the proportion of potential host-specific proteins encoded in the genome of *S. solidus*.

#### Pipeline B - host-parasite specific protein similarity analysis

**Figure 2:**
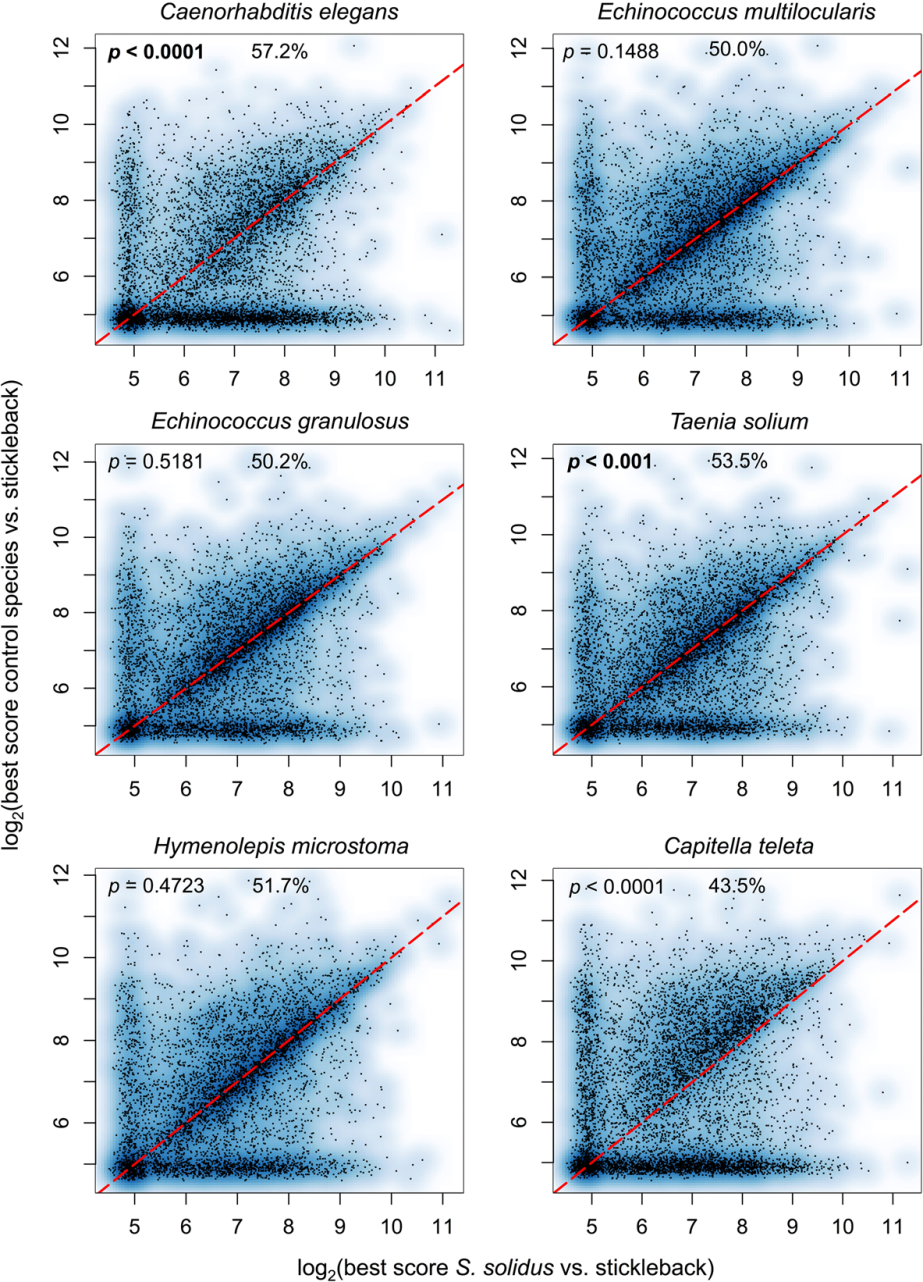
Protein similarity comparisons using full-length *blastp* searches against various parasitic and non-parasitic control worm species. Scatter plots of the best *blastp* scores of *S. solidus* proteome (x axis) and six control worm species proteomes (y axis, name of the species specified on top of each graph) against the proteome of the threespine stickleback. Data below the red dotted line correspond to stickleback proteins showing a higher sequence similarity for *S. solidus* as compared to the corresponding control worm species. For each scatter plot, a Wilcoxon signed-rank test was performed on the distributions of *blastp* scores (*p-value* in the upper left corner of each graph). Significant *p-values* were highlighted in bold for higher *blastp* scores between *S. solidus* and its host than between the control species and the host. The percentage of the stickleback proteome showing higher scores with *S. solidus* than with the control species appears on top of each scatterplot.

Identification of candidate mimicry proteins was achieved by the use of an iterative sequence similarity approach designed to identify highly similar peptides between a parasite and its host (pipeline B in Figure 1, adapted from the method described in [11]). We used custom Python scripts (https://github.com/fohebert/Scripts.git) to adapt the original idea from Ludin et al. [11] to our study system, as presented in Figure 2 (pipeline B). First, sequence similarity between the *S. solidus* proteome and several control proteomes (Table 1) was assessed by using ungapped *blastp* searches. Proteins returning an e-value ≤ 1e-15 were discarded, as they were considered generally conserved among eukaryotes. Proteins exhibiting low similarity with control proteomes were then analyzed using PHOBIUS to identify potential N-terminus signals. Again, these short and conserved sequences were trimmed out using our custom-made Python scripts designed for pipeline A. Trimmed sequences were subsequently fragmented in overlapping 14-mers, following the same procedure used in the first pipeline (Figure 1). These *S. solidus* 14-mers were blasted against the complete array of control proteomes and sequences above pre-defined identity thresholds (as empirically determined and validated in [11], see red solid line, Additional file 2) were discarded. Remaining *S. solidus*-specific peptides (i.e. 14-mers that share very low or no similarity with control proteomes) were screened against the host proteome with an ungapped *blastp* search. These peptides were also screened against five non-host fish proteomes (*Danio rerio, Xiphophorus maculates, Oryzias latipes, Lepisosteus oculatus* and *Takifugu rubripes,* see Table 1), all sharing a distant common ancestor with the stickleback [63-65] and for which no infection by *S. solidus* has ever been reported due to the high specificity of the infection [28]. This extra step was performed in order to assess the proportion of candidate 14-mers expected by chance only when investigating any fish proteome, although we cannot exclude that mimicry proteins might resemble proteins generally conserved among fish taxa. Queries returning hits with sufficient similarity were labeled as molecular mimicry candidates. GoMiner [66] was ultimately used to perform an enrichment analysis for gene ontology terms on these final sequences to identify potential biological functions over-represented among candidates. Final candidates were also screened for the presence of a secretory signal peptide using SignalIP [67] to label proteins as secreted or non-secreted. As an additional control to assess the statistical significance of the method, we performed the same analysis (i.e. pipeline B) on a randomized version of the proteome of *S. solidus* using EMBOSS function *shuffleseq* [11,55]. This second pipeline is thus similar to pipeline A, although it does not include other parasite proteomes. By doing so, proteins potentially involved in convergent molecular mechanisms of phenotypic alteration will not be discarded by comparing *S. solidus* proteins to other parasite proteins [68]. Pipeline A thus served as a first exploratory phase that allowed us to classify proteins according to their level of conservation across a wide range of phylogenetic groups, whereas pipeline B was specifically used to identify mimicry candidates between *S. solidus* and the threespine stickleback.

#### RT-PCR validation

We further confirmed that our refined set of candidates identified through pipeline B were expressed by the parasite by performing a reverse transcription polymerase chain reaction (RT-PCR) on 17 additional worms from a different population than the one used to build the transcriptome and from three different developmental stages, i.e. pre-infective (n = 7), infective (n = 7, > 50 mg) and post-reproduction adults (n = 3, > 350 mg) in a simulated bird gut. Worms were extracted from a population of lab-raised and artificially infected sticklebacks at the University of Leicester, England (UK). Details of the method and complete primer sequences are available in Additional file 3.

#### Host contamination control

To eliminate potential fish cDNA contamination that could have been introduced in the transcriptome due to host tissues inadvertently left on the parasite’s integument during the dissection, three different bioinformatics controls were used. First, cleaned reads were mapped to the raw genome of *Schistocephalus solidus* using BWA-SW [69] with default parameters. Default parameters for the program BWA are designed to offer the best possible balance between performance and accuracy. The program also automatically adjusts parameters according to read length and error rates [60]. BWA default parameters are thus sufficiently efficient to achieve the goal of discarding low quality reads that do not match the reference genome. Reads that did not map on the genome were discarded from the assembly. The second control was performed at the end of the assembly. Final contigs obtained with both assembly methods (RAY and MIRA) were blasted against the raw genome of *S. solidus* and sequences returning no hits were discarded. A third control was finally used to confirm that the mimicry candidates found with pipeline B (Figure 2) were tapeworm proteins and not cDNA contamination from the host. Candidate proteins were blasted against the proteomes of the host and the parasite (*blastp* searches) and cDNA sequences corresponding to these proteins were also respectively blasted against the genomes of the host and the parasite (*blastn* searches), which allowed us to assess if these sequences (at the levels of nucleic acids and amino acids) were more similar to the tapeworm or the fish proteome. When the e-value, the raw bit score and the length of alignment were systematically greater when blasted against the parasite as compared to the host, candidates were considered as true parasite sequences and not contamination. We also performed a fourth control in the laboratory to determine whether the four candidate mimicry genes do originate from *Schistocephalus solidus* and not from threespine stickleback DNA contamination. To do so, we conducted a PCR validation experiment using DNA from three pools of coracidia, the free swimming stage of *Schistocephalus solidus* that has never been in contact with the fish host, where each pool came from one breeding pair. We also used genomic DNA (gDNA) and cDNA samples from three individual adult worms and host fish (Additional file 3).

### Ethical approval

All animal experiments that were performed at the Max Planck Institute for Evolutionary Biology (Plön, Germany) were approved by the ‘Ministry of Energy, Agriculture, the Environment and Rural Areas’ of the state of Schleswig-Holstein, Germany (reference number: V 313–72241.123-34). Fish were captured under U.K. Environment Agency permit and with the permission of the landowner. All animal experiments performed at the University of Leicester (England, UK) were undertaken under a U.K. Home Office license (PPL80/2327), in accordance with local and national regulations, and in line with ABS/ASAB guidelines for the ethical treatment of animals in behavioral research (available online at http://asab.nottingham.ac.uk/ethics/guidelines.php).

## Results and discussion

### Transcriptome assembly

The multiple assembly strategy used in this study yielded a total of 9,093 putative protein-coding genes (Table 2, Additional files 4 and 5), which is slightly lower than expected based on recent flatworm genome assemblies (between 10,231 and 12,490 genes, see [54]) but on the same order of magnitude. Two plausible reasons could explain this discrepancy: i) not all life stages and hosts were sampled (earlier larval stages and adult missing) and ii) a relatively low median coverage (average: 7X, median: 6.93X) could have resulted in the elimination of “true gene” transcripts which have insufficient coverage. However, since there is no published consensus transcriptome or complete genome annotation for *S. solidus*, the number of genes and these explanations remain speculative and we consider our predicted proteome dataset (referred to as proteome) to be a conservative estimate. On the other hand, the most relevant developmental stage required to answer the main question asked in this study (nine weeks post-infection, when behavioral changes in the host would be apparent) was sampled. Relevant genes involved in molecular mimicry at this stage and in the fish are therefore likely to be contained in the dataset used to perform our analyses.

**Table 2:** **Transcriptome assembly metrics**

Total number of reads	528 595
*Number of reads mapping on raw genome* ^*1*^	471 018 (89%)
*Assembled reads*	462 128
Number of gene-contigs	9093
Average length in bp (range; median)	1266 (212 – 7588; 1097)
*N50*	945
*Average coverage (range; median)*	7.03X (4 – 1850; 6.93)
Average protein length in amino acids (range; median)	300 (20 – 1992; 258)
Number of annotated proteins^2^	5949

^1^Genome available from 50 Helminth Genomes Initiative (http://sanger.ac.uk/).

^2^Based on swissprot database.

### Comparisons with full-length proteins

Results from a first glance at the dataset, using full-length *blastp* searches among various parasitic and non-parasitic worms, confirmed the potential for candidate mimicry identification among *S. solidus*’ proteins (Figure 2). Full-length *blastp* searches on the proteomes of *S. solidus* and six other worm species (four of which are cestodes and two are non-parasitic free living worms) revealed various levels of protein similarities depending on the species being compared to *G. aculeatus*. When *blastp* scores between *S. solidus* and its host were compared to *blastp* scores between *C. elegans* and *G. aculeatus*, for a given host protein, on average, *S. solidus* shared a significantly higher blast score (*p* < 0.0001, two-tailed Wilcoxon test, Figure 2). This trend was also true when the association *S. solidus*-stickleback was compared to the association *T. solium*-stickleback, i.e. stickleback proteins were, on average, more similar to *S. solidus*’ proteins than *T. solium*’s proteins (*p* < 0.001, two-tailed Wilcoxon test, Figure 2). However, when the comparison is performed with any of the three other cestodes, there is no significant difference between distributions of *blastp* scores (*p* = 0.1488 for *S. solidus* vs. *E. multilocularis*, *p* = 0.5181 for *S. solidus* vs. *H. microstoma*, *p* = 0.4723 for *S. solidus* vs. *E. multilocularis*, two-tailed Wilcoxon tests, Figure 2). When the proteome of *C. teleta*, a non-parasitic marine polychaete, was blasted against the stickleback proteome, we observed significantly higher blast scores between the two species, as compared to the scores obtained between *S. solidus* and the stickleback (p < 0.0001, two-tailed Wilcoxon test, Figure 2). This could be due to the fact that the proteome of *C. teleta* contains a higher number of proteins than the proteome of *S. solidus* (32 175 and 9 093 respectively). It is thus expected that by chance alone, more similarities can be found when the *blastp* search against the stickleback proteome is performed with a larger set of proteins.

### Insights from a general parasitic protein similarity analysis (pipeline A)

After this first round of full-length *blastp* searches, predicted *S. solidus* proteins were analyzed through pipeline A. Predicted *S. solidus* proteins were fragmented and compared to several other proteomes (see Methods, Table 1), which provided a general classification of protein similarity among various phyla (Figure 1, pipeline A). In total, 8,786 proteins passed the similarity thresholds, returning significant hits on various species. Most of these proteins were widely distributed among phyla (total = 86%; controls & parasites = 4877 proteins, 53%; controls & parasites & host = 2981 proteins, 33%), while small proportions were assigned to a given group only (controls = 338, 3.7%; parasites = 562, 6.2%). Based on empirically determined thresholds (Additional file 2, see [11]), only three *S. solidus* proteins showed a high degree of sequence similarity exclusively with host proteins: tektin-4, partial coding sequence from jockey-like mobile element, unknown predicted protein. According to pipeline A, sequences falling into this category were deemed the most interesting candidates for molecular mimicry, i.e. short peptides showing strong homology to a corresponding host protein (between 85% and 100% sequence similarity). However, none of these sequences had a gene identifier that could directly associate them to a molecular mimicry strategy. One of these candidate proteins is similar to tektin-4 [UniProt:GAA27704], which is involved in microtubule cytoskeleton organization, therefore not secreted or expressed at the cell surface. Another candidate corresponds to a polymerase from a mobile element [UniProt: CCD80178], thus not involved in any physiological process or biological function that would relate to the host phenotype. The last candidate, a relatively short unknown protein (73 amino acids) shares only 11 identical residues with an unknown stickleback protein (275 amino acids, Ensembl:ENSGACT00000002277). Since no function can be established based on current information, either for the tapeworm peptide or for the host target, this last candidate will require further studies in order to confirm its role in host behavioral changes.

### Identification of mimicry peptides through pipeline B

**Figure 3:**
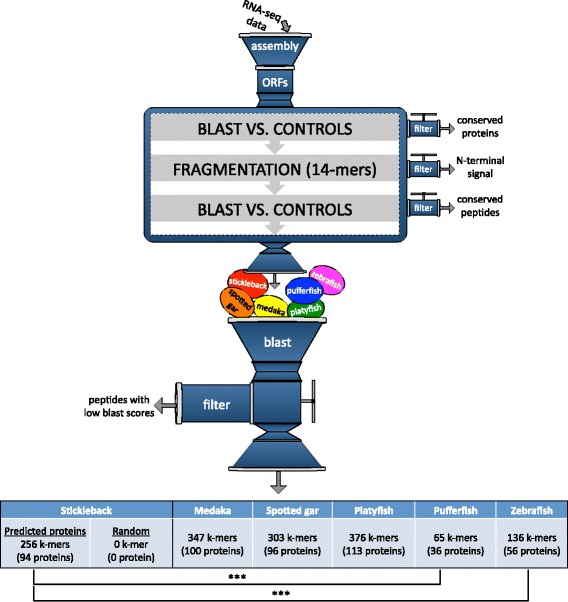
More than 200 tapeworm peptides were identified as significant mimicry candidates through *in silico* pipeline B. Candidate mimicry identification was achieved through protein similarity analyses. Conserved proteins among distantly related species were discarded based on their high raw similarity score against five different control proteomes, while very similar peptides considered as mimicry candidates were kept based on their high raw similarity score against the stickleback (“true host”) or any of the non-host control (*D. rerio*, *T. rubripes*, L*. oculatus*, *O. latipes*, and *X. macalatus*). *S. solidus* shuffled proteins were also analyzed through pipeline B to make sure that no mimicry peptide could be found only by chance, even with “non-functional biological sequences”. ***p < 0.0001.

In this last sequence similarity analysis performed on *S. solidus* proteome, predicted proteins were fragmented again and compared with control and host proteomes only (see Methods, Figure 1, pipeline B), allowing the identification of very specific peptides potentially involved in the phenotypic changes observed in parasitized sticklebacks. In this analysis, as in pipeline A, peptides (14-mers) were generated based on full-length protein sequences. However, in this second analysis, more stringent criteria were used and this time, tapeworm proteins were not compared to proteomes from vertebrate parasites (see Methods for details). The complete predicted proteome dataset for *S. solidus* (9,093 proteins) was fragmented in 1,240,045 overlapping 14-mer peptides that were successively compared with all control species according to pipeline B (see Methods, Figure 1). After discarding conserved proteins and peptides, empirically determined similarity thresholds with the stickleback proteome allowed the identification of 287 candidate 14-mers (0.023% of all 14-mers) distributed in 94 proteins (Additional file 6). More than half of these candidate peptides (167) shared 100% similarity (i.e. 14 identical residues) with a host sequence. Based on the third control for host cDNA contamination (see Methods), we confirmed that these 14-mer candidates are part of true tapeworm proteins, as these proteins (i.e. proteins containing the candidate peptides) were always more similar to *S. solidus* proteins or any other closely related parasites (e.g. cestodes; *Echinococcus granulosus*, *Hymenolepis microstoma*, trematodes; *Clonorchis sinensis*, *Opisthorchis viverrini*, *Schistosoma mansoni*) than any fish protein (full-length *blastp* searches). We tested if these proteins were enriched for particular biological functions using GoMiner [66]. No gene ontology (GO) category was over-represented among these candidates (data not shown). To assess the statistical relevance of the candidate 14-mers, the same procedure was carried out with a randomized version of the *S. solidus* proteome. The rationale behind this test is that finding as many mimicry candidates with a randomized version of the proteome as in the predicted proteome would mean that our results have no biological meaning. Shuffled sequences did not return any candidate, thus underlining the potential significance of the candidate 14-mers identified among real sequences (Figure 3).

A final control for the method was performed using fishes that are usually not infected by *S. solidus* in the wild. This additional analysis acts as a validation step aimed at testing the method in a non-specific context, i.e. when parasite-specific peptides are screened against proteins from non-host species (see Methods). By successively screening parasite-specific peptides against five different non-host fish proteomes through pipeline B, we found 136 (in 56 proteins), 303 (in 96 proteins), 347 (in 100 proteins), 65 (in 37 proteins) and 376 (in 113 proteins) candidate mimicry peptides when using *D. rerio*, *L. oculatus*, *O. latipes*, *T. rubripes* and *X. maculatus,* respectively (Figure 3). At the peptide level, we found significantly more mimicry candidates for non-host species when screened against *O. latipes* and *X. maculatus* as compared to the stickleback (*p* = 0.0172 and *p* < 0.0001 respectively, 2-sample tests of equal proportions). On the other hand, we found no significant difference between the numbers of mimicry candidates identified with the stickleback versus *L. oculatus* (*p* = 0.51, 2-sample test of equal proportions). Results also indicate that significantly more mimicry peptides can be found when screening against the stickleback as compared to screening against *D. rerio* or *T. rubripes* (*p* < 0.0001 for both screens, 2-sample tests of equal proportions). When looking at the protein level, we found no significant difference between numbers of candidate mimicry proteins identified with non-hosts *L. oculatus*, *O. latipes*, and *X. maculatus* as compared to the real host (*p* = 0.885, *p* = 0.885, *p* = 0.189 respectively, 2-sample tests of equal proportions). Finally, we found significantly more candidate mimicry proteins when screening against the real host than when screening against either non-hosts *D. rerio* or *T. rubripes* (*p* < 0.0001, 2-sample tests of equal proportions, see Figure 3).

Overall, similar numbers of candidate mimicry proteins can be found when using either the real host or three out of five non-host fishes (i.e. *L. oculatus*, *O. latipes*, and *X. maculatus*). If *S. solidus* uses a molecular mimicry strategy to complete its life cycle, we can assume that the mimicry proteins produced by the parasite will be very similar to fish proteins (if this is a case of mimicry created by sequence similarity, whereas three-dimensional structural, as well as functional mimicry [70] cannot be identified using this method). Finding similar results when screening against non-host fishes as compared to the real fish host may not be surprising, considering that the mimicry strategy can be targeting any common pathway found in fishes (or vertebrates). Moreover, since we did not use any fish or vertebrate proteome among the control group for pipeline B, proteins and peptides highly conserved across vertebrate species were kept throughout the analyses. We can thus assume that some of the mimicry peptides identified when screening against non-host fishes reflect peptide/protein conservation across fish or vertebrate taxa. Results suggest that this mimicry identification method can efficiently isolate high profile candidates. However, it cannot assess their true biological role in the interaction between the parasite and its host as functional studies are required to perform this task.

### Secreted proteins: the most plausible candidates

*Schistocephalus solidus* is an endoparasite living in the abdominal cavity of its host. As a consequence, if it uses mimicry proteins to affect physiological and cellular pathways in its host, the “phenotype-altering signal” is most likely to come from a protein that is secreted by the parasite and released in the bodily fluids of the host or that is expressed at the cell surface. Strong candidates for molecular mimicry should thus be secreted and/or membrane proteins. Among all proteins containing 14-mer candidates, only four could be labeled as secreted or cell-surface localized (i.e. containing a secretory signal peptide) based on SignalIP results (see Methods, Table 3 & Additional file 7). Interestingly, one of these candidates belongs to the WNT proteins family (14-residue homology), WNT4 [Uniprot:A0A068WB45], a large group of signaling molecules involved in cell-to-cell interactions and various developmental pathways [71]. This candidate is also very similar to the protein WNT5B (found in the fish *Danio rerio*, Uniprot:NP_571012), which plays a role in the development of discrete regions of tissues. Previous proteomics investigations found that molecules from the Wnt family were over-expressed in the head of crickets and grasshoppers infected by a behavior-altering hairworm [21,22]. Specifically, protein fragments from the Wnt family involved in the central nervous system (CNS) development and produced by the parasite were found in infected orthopterans exhibiting abnormal suicidal behaviors. Even though the WNT4 protein identified in our study is not known to be directly involved in the development of the CNS in fishes, the common point between these different host-parasite systems could be a general disruption of cell-to-cell communication, leading to various changes in behavior [23,72]. Another *S. solidus* peptide matched to a membrane zinc transporter [Uniprot:Q504Y0] (14-residue homology) suspected to be involved in the development of schizophrenia in humans [73]. Its biological function in *S. solidus* or as a mimicry protein in its fish host remains to be investigated. Two other parasite-specific 14-mers showed high similarity (19-residue homology) with a zinc finger protein responsible for a palmitoyltransferase activity in the stickleback [ZDHHC18, Ensembl: ENSGACT00000009617]. Palmitoylation represents one of many different types of post-translational modifications of proteins. Specifically, it involves the addition of the palmitate lipid in a thioester linkage on cysteine residues [74,75]. It has been shown that palmitoylation of neuronal proteins like PSD-95 in humans can lead to changes in synaptic plasticity, thus potentially changing the way the information is transmitted throughout the central nervous system [76]. While empirical evidence supports the role of these lipid modifications in the dynamic regulation of protein function and neuronal signaling [76], no direct link can yet be established between this protein and phenotypic changes in the stickleback. Overall, pipeline B allowed us to identify significant candidates with potential roles in cell signaling or cognitive pathways, but their direct impact on host phenotypes and their level of implication in the host-parasite molecular cross talk cannot be assessed unless functional studies are carried out.

**Table 3:** **Secreted proteins identified as mimicry candidates through pipeline B**

**Parasite protein**	**No. of 14-mers with significant BLASTp results**	**Ave. Identity**	**GO terms**	**Protein ID** ^**1**^	**Function** ^**2**^
				**(Access. no)**	
Ssc1854	1	12/14	Ion transport;	Zinc transporter, ZIP12 (Q504Y0)	Zinc influx transporter.
			Zinc transport		
Ssc2726	2	12/13	Transcription regulation;	Lysyl oxidase homolog 2B,	Mediates the post-translational oxidative deamination of lysine residues
			Transcription	LOXL2B (A1L1V4)	
Ssc5533	2	12/14	Ion transport;	Palmitoyltransferase, (ZDHCC17) EUB64135	Transferase activity. Lipid modification activity involved in protein trafficking and function in the central nervous system.
			Transport		
Ssc6185	1	13/14	Wnt signaling pathway	Protein WNT4	Signaling molecule possibly involved in the development of discrete regions of tissues.
				A0A068WB45	

^1^Best blast hit when screened against Uniprot (http://uniprot.org).

^2^Information on function taken from Uniprot.

We also confirmed that these four mimicry candidates originate from the parasite and not from host DNA contamination (see Methods & Additional file 3: Figure S1). Our results showed that each candidate gene produced an amplification product for all parasite DNA templates (coracidia gDNA, adult gDNA, and adult cDNA) but not for fish DNA samples, except *palmitoyltransferase* which only amplified in gDNA with a larger than expected amplicon size (Additional file 3: Figure S1). *Wnt04*, *palmitoyltransferase* and *lysyl oxidase homolog 2B* produced the expected length of PCR products (approximately 190 bp, 180 bp and 170 bp, respectively) for the different types of parasite DNA templates. However, the amplicon for *membrane zinc transporter* was larger in parasite gDNA (approximately 280 bp) than in parasite cDNA samples (160 bp), perhaps because the primer pair used to amplify this gene spans an intron or because of alternative splicing events. By performing this additional control, we were able to confirm the absence of host DNA contamination in our final candidates.

### RT-PCR validation

**Figure 4:**
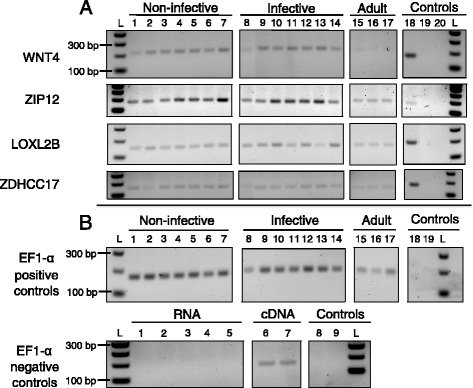
Expression of mimicry candidate genes in three different *S. solidus* developmental stages confirmed by retrotranscription polymerase chain reaction (RT-PCR). RT-PCR was performed on 17 different *S. solidus* worms from three life stages (non-infective, infective and post-reproduction adults) to confirm that the four best mimicry candidate genes, i.e. secreted proteins, are expressed in at least one developmental stage of the parasite. **A**) Gel electrophoresis for each gene, wnt4 (WNT4), zinc transporter (ZIP12), lysyl oxidase (LOXL2B) and palmitoyltransferase (ZDHCC17). Wells 1–7 contain cDNA from non-infective worms (mass < 50 mg), wells 8–14 contain cDNA from infective worms (mass > 100 mg) and wells 15–17 contain cDNA from adult worms (mass > 350 mg, after egg production outside the fish). Well 18 = positive control with elongation factor 1 alpha (EF1-α), a gene commonly expressed in all tissues and developmental stages. Well 19 = negative control (no template). Well 20 = negative control (no primers). **B**) Additional controls to confirm that our positive control gene (EF1-α) is expressed in all worms and all life stages and to confirm that RNA samples used to perform the RT-PCR reactions are DNA-contamination free. Positive control: non-infective stage (wells 1–7), infective stage (wells 8–14), adult stage (wells 15–17) and controls (well 18 = no template, well 19 = no primers). Negative control: wells 1–7 (RNA samples from all three life stages, i.e. two non-infective, two infective and one adult worms respectively), wells 6–7 (positive controls with cDNA from one infective worm and one non-infective worm respectively), wells 8–9 (negative control, no template and no primers respectively).

Bearing in mind the importance of validation when it comes to results obtained through complete *in silico* methods, we conducted an additional analysis on parasites bred in the laboratory to confirm that our candidate genes are expressed in different developmental stages (see Methods & Additional file 3). We sampled 17 additional parasites, performed RNA extractions on whole worms, designed and tested primers and performed RT-PCR reactions on all worms. Results indicate that the four candidate genes are expressed in three developmental stages spanning the entire growth period within the fish host and this holds true for all of the additional worms sampled (Figure 4). Such empirical evidence confirms the expression of the candidates in different life stages. However, RT-PCR data is used as a test of presence/absence of expression and does not quantify at which level each gene is expressed in each worm life stage. Small differences in expression levels among developmental stages (i.e. not detectable through the validation method we used) could produce variable phenotypes depending on the background physiological state and the expression levels of other non-studied genes. Secreted proteins identified in this study as potential candidates for molecular mimicry exhibit consistent expression throughout key stages associated with host phenotypic alterations, which prompts the development of follow-up studies.

## Conclusion

In this study, we identified mimicry candidate peptides from a behavior-altering cestode that showed high sequence similarity with specific host proteins. Two different *in silico* pipeline analyses were built and used to identify these candidates, which acts as useful analytical tools that can be used in any host-parasite system to perform the same task. The expression of the candidate protein-coding genes in three developmental stages of the parasite was also confirmed by RT-PCR, thus confirming their importance throughout *S. solidus’* life cycle. Candidates identified through these analyses were selected based on sequence similarity only and should not be considered as evidence for any mechanistic link between infection and phenotypic changes in physiology and behavior. Further proteomics and transcriptomics analyses as well as functional assays in different life stages of the parasite and in uninfected fish should help understand the role of these candidate proteins during the infection of the stickleback and reinforce our knowledge on the molecular bases of complex ecological interactions taking place between a parasite and its host.

## Additional files

Additional file 1: Figure S1. CD-HIT-EST parameters used in the *de novo* assembly process. CD-HIT-EST uses an incremental greedy algorithm that sorts sequences in order of decreasing length. The longest sequence becomes the “representative sequence” (R) of the first cluster. All other sequences are then compared to the representative sequence of each cluster. If similarity with the representative sequence is above a certain threshold, the sequence is added to the cluster, otherwise it becomes the representative sequence of a new cluster. In our analysis, the shorter sequences (S) had to cover at least 85% of the length of the representative sequence (s = 85%). Alignment coverage threshold on the representative sequence (aL) was set to 85% and alignment coverage threshold on other sequences in the cluster (aS) was also set to 85%. Sequence similarity threshold was 90%. R_a_ = portion of the representative sequence (R) that aligns with all other sequences in the cluster. S_a_ = portion of the shorter sequence (S) that aligns with all other sequences in the cluster. See http://weizhong-lab.ucsd.edu/cd-hit/wiki/doku.php?id=cd-hit_user_guide for further details. Additional file 2: Figure S2. Ungapped BLAST-p identity thresholds used in this study. Thresholds were empirically determined by Ludin et al. [11] and confirmed in this study after performing several tests. Red line: conserved proteins thresholds, queries showing identities below the line (values in red) were considered as “conserved” and were discarded. Green line: high similarity thresholds, queries showing identities above the line (values in green) were considered as potential mimicry candidates. Additional file 3:
**Supplementary methods.** Detailed description of the methods used to confirm, by RT-PCR and simple PCR, that the mimicry candidate genes identified in this study are expressed in different life stages of *S. solidus* and that no host contamination can be found in our dataset [77,78]. Additional file 4:
**Final DNA sequence assembly.** Complete set of DNA sequences obtained after performing combined *de novo* assemblies. This cleaned and annotated sequence dataset represents the reference transcriptome used in this study to predict tapeworm proteins and perform the *in silico* mimicry pipelines. Additional file 5:
***Schistocephalus solidus*** **predicted proteome.** Complete set of ORFs predicted from our reference transcriptome. Mimicry protein and peptide identification was performed on this dataset. Additional file 6: Table S1. 14-mer mimicry candidates. Complete set of 14-mer mimicry candidates as identified through pipeline B. Additional file 7:
**Secreted Protein/peptide mimicry candidates.** Protein and DNA sequences for the four final candidates labeled as secreted proteins and identified through pipeline B.
