# Supplementary figures and images for: Identification of candidate mimicry proteins involved in parasite-driven phenotypic changes
Source: Parasit Vectors. 2015 Apr 15;8:225. doi: 10.1186/s13071-015-0834-1 (PMC4407394; doi:10.1186/s13071-015-0834-1)

Sequence similarity threshold = 90%

$$s = S_a/R_a = 85\%$$

$$aL = R_a/R = 85\%$$

$$aS = S_a/S = 85\%$$

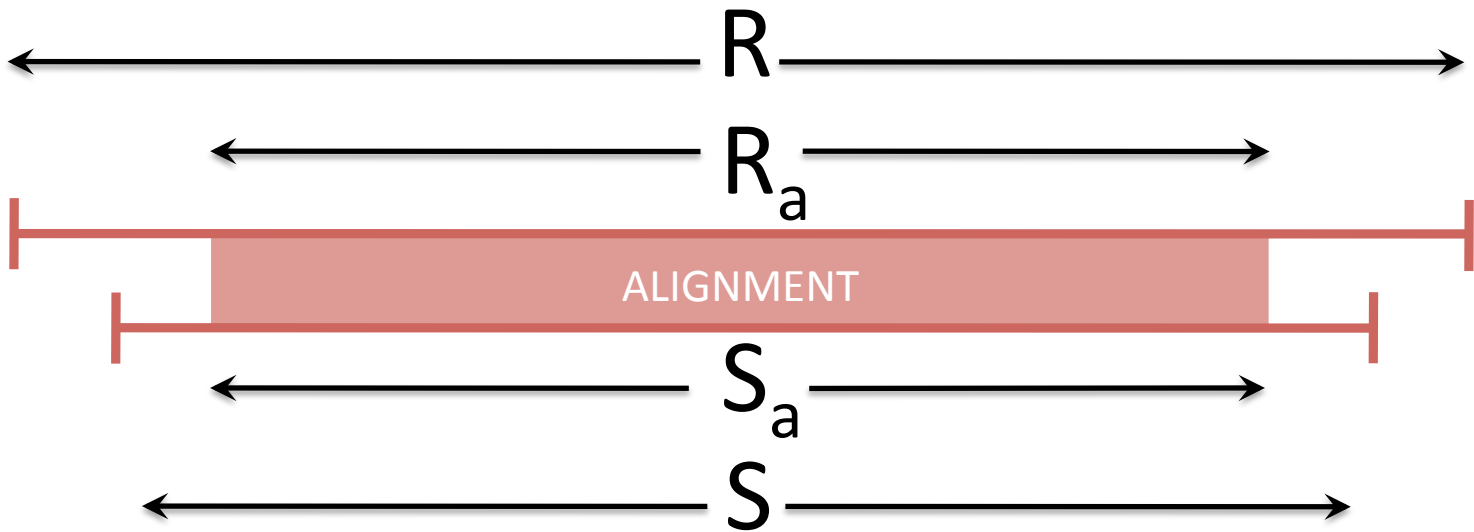

Supplement: Additional file 1: Figure S1. — CD-HIT-EST parameters used in the de novo assembly process. CD-HIT-EST uses an incremental greedy algorithm that sorts sequences in order of decreasing length. The longest sequence becomes the “representative sequence” (R) of the first cluster. All other sequences are then compared to the representative sequence of each cluster. If similarity with the representative sequence is above a certain threshold, the sequence is added to the cluster, otherwise it becomes the representative sequence of a new cluster. In our analysis, the shorter sequences (S) had to cover at least 85% of the length of the representative sequence (s = 85%). Alignment coverage threshold on the representative sequence (aL) was set to 85% and alignment coverage threshold on other sequences in the cluster (aS) was also set to 85%. Sequence similarity threshold was 90%. Ra = portion of the representative sequence (R) that aligns with all other sequences in the cluster. Sa = portion of the shorter sequence (S) that aligns with all other sequences in the cluster. See http://weizhong-lab.ucsd.edu/cd-hit/wiki/doku.php?id=cd-hit_user_guide for further details. [file 13071_2015_834_MOESM1_ESM.pdf]

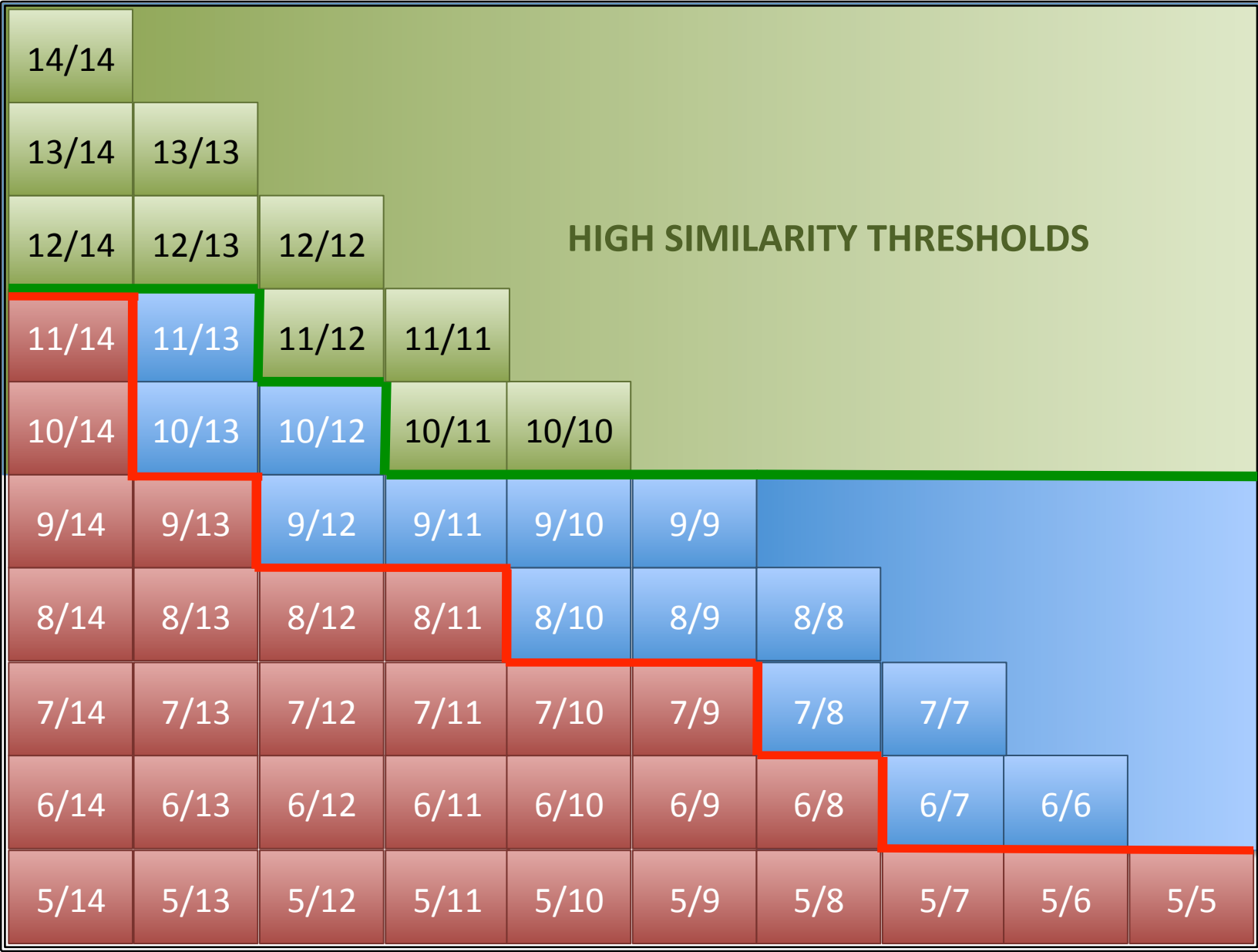

Supplement: Additional file 2: Figure S2. — Ungapped BLAST-p identity thresholds used in this study. Thresholds were empirically determined by Ludin et al. [11] and confirmed in this study after performing several tests. Red line: conserved proteins thresholds, queries showing identities below the line (values in red) were considered as “conserved” and were discarded. Green line: high similarity thresholds, queries showing identities above the line (values in green) were considered as potential mimicry candidates. [file 13071_2015_834_MOESM2_ESM.pdf]
