# Supplementary material for: Identification of candidate mimicry proteins involved in parasite-driven phenotypic changes
Source: Parasit Vectors. 2015 Apr 15;8:225. doi: 10.1186/s13071-015-0834-1 (PMC4407394; doi:10.1186/s13071-015-0834-1)
Supplement: Additional file 3: — Supplementary methods. Detailed description of the methods used to confirm, by RT-PCR and simple PCR, that the mimicry candidate genes identified in this study are expressed in different life stages of S. solidus and that no host contamination can be found in our dataset [77,78]. [file 13071_2015_834_MOESM3_ESM.pdf]

Supplementary methods

Contamination control – PCR validation

Genomic DNA from adult worms (3 - 5 mm tissue) and gDNA from the tailfin of threespine sticklebacks were extracted using Qiagen DNeasy Kit (Hilden, Germany). Coracidia DNA was extracted using Chelex® (Bio-Rad, Hercules, California) following the protocol of Dolnik *et al*. (2009). RNA from adult worms and the head kidney of the threespine sticklebacks were extracted using the NucleoSpin RNA kit (Macherey-Nagel). The synthesis of cDNA was performed using Omniscript Reverse Transcriptase (Qiagen) according to the manufacturer's protocol.

PCR amplifications were prepared in a 25 μl reaction volume containing 50 - 100 ng template DNA, 2 U Taq polymerase (Invitek), and a final concentration of 0.05mM dNTPS (Metabion), 4.5 mM $MgCl_2$ (Invitek), 1x NH_4MgCl_2-free Buffer (Invitek), and 0.2 μM each of forward and reverse primers. The amplification profile consisted of an initial denaturation for 15 min at 95°C followed by 35 cycles of denaturation at 94°C for 30 sec, annealing for 90 sec at an appropriate temperature, and extension at 72°C for 1 min with a final extension time of 5 min at 72°C. The different primer pairs used were Ssc1854F 5'-GGAGAACTCCCAGTATCATGCC-3' and Ssc1854R 5'- CGGCTTGTCCACCGCATTG-3' for *membrane zinc transporter*, Ssc2726F 5'-CTCACGAACGCAACGACATC-3' and Ssc2726R 5'-GTAGTTGCCTGCAGAGCGTA-3'for *lysyl oxidase homolog 2B*, Ssc5533F 5'-CTCACGAACGCAACGACATC-3' and Ssc5533R 5'-GTTGATAGCGGCCCAGTGTA-3' for *palmitoyltransferase*, and Ssc6185F 5'-TAAGGAGTACGGCTTCGCAC-3', and Ssc6185R 5'-CTTGGAGAATCGGTAGGCGT-3' for *Wnt04*. These primers were designed based on the nucleotide transcript sequences of the four candidate *Schistocephalus solidus* mimicry genes (Additional file 7).

Sampling of additional worms for RT-PCR validation

Additional worms were sampled from a population of lab-raised threespine stickleback in Leicester University, England (UK). More specifically, 17 worms were extracted from the abdominal cavity of their host, among which seven were non-infective (parasite mass < 50 mg), seven were infective (parasite mass > 50 mg) and three were cultured in nutritive medium (following the method described in [77]) until egg production (parasite mass > 300 mg, post-reproductive stage). Worms were cut into small pieces of 0.5 cm by 0.5 cm and individually placed in a 1.5 mL eppendorf tube filled with RNA later (1 mL). RNA was extracted using TRIzol ® reagent (Life Technologies, Burlington, ON, Canada) following the manufacturer's protocol.

Validation of candidate gene expression using RT-PCR

Primer design

Primers were designed for each of the four candidate genes identified through pipeline B (see Figure 1). We also designed primers for a common "house-keeping gene" in tapeworms [78], i.e. elongation factor-alpha 1 (EF1-α), that we used as a positive control in each of the RT-PCR performed (see Table S1 below). For primer design, these general guidelines were followed: amplicon size between 120-220 bp, annealing temperature between 56-62°C (no more than 5°C difference between forward and reverse), GC content of 55%±5 and amplicon located close to the 3' end of the gene.

DNase treatment

Each RNA sample was treated with DNase I (amplification grade 1 U/µL, Life Technologies, Burlington, ON, Canada) prior to the RT-PCR reaction to eliminate potential DNA contamination: 1 µL DNase I + 1 µL 10X DNase I reaction buffer + 5 µL of diluted RNA 200 ng/µL + 3 µL nuclease-free H_2O (final volume = 10 µL) incubated at room temperature for 15 minutes, after which 1 µL of 25 mM EDTA solution was added to every sample for a 10-minute incubation period at 65°C.

Retro-transcription

Retro-transcription was performed using 1 µL of dNTP mix (10 mM), 1 µL of primer stn (oligo dT 500 ng/µL + random hexamers 100 ng/µL, Life Technologies, Burlington, ON, Canada), 4 µL of DNase-treated RNA (100 ng/µL) and 2 µL of nuclease-free H_2O (final volume = 12 µL). Final solution was incubated at 65°C for 5 minutes and then 4°C for 10 minutes. Each sample was incubated at 42°C for 2 minutes with a reaction solution made with 4 µL of 5X Superscript II buffer (Life Technologies, Burlington, ON, Canada), 2 µL of DDT (0.1M) and 1 µL of RNase out recombinant ribonuclease inhibitor (Life Technologies, Burlington, ON, Canada) for a final volume of 7 µL per DNase-treated RNA sample. After this short incubation perdiod, 1 µL of Superscript II reverse transcriptase (Life Technologies, Burlington, ON, Canada) was added to each sample and incubated according to the following cycle: 25°C for 10 minutes, 42°C for 60 minutes and 70°C for 15 minutes (4°C “forever” after completion of the cycle).

RT-PCR reaction

RT-PCR reactions were performed using an Eppendorf-Mastercycler ® EP gradient S system (Eppendorf, Mississauga, Ontario, Canada) with the following reaction solution: 13.5 µL of nuclease-free H_2O, 1 µL of dNTPs (10 mM), 0.25 µL of TAQ polymerase (5 U/µL, Life

Technologies, Burlington, ON, Canada), 2.5 µL of 10X TAQ buffer (with $MgCl_2$, Life Technologies, Burlington, ON, Canada), 5 µL of diluted cDNA (1:50) and 2.5 µL of primer mix (forward + reverse), in a final reaction volume of 25 µL per sample. A reverse transcription negative control (no reverse transcription) was performed on five different worms spanning the three life stages (n = 2 for pre-infective, n = 2 for infective and n = 1 for sexually mature adult) to confirm the absence of gDNA contamination. Two additional negative controls for each gene run were performed: one with no template (to confirm the absence of primer contamination) and one with no primer (to confirm that there is no cDNA contamination that would cause unwanted amplification). RT-PCR was performed following these conditions: 92°C for 2 minutes, 95°C for 20 seconds, 56°C or 58°C (depending on primer Tm) for 40 seconds, 72°C for 1 minute (30 cycles), 72°C for 10 minutes and finally 4°C "forever". PCR products were analyzed by electrophoresis on 2% agarose gel.

Table S1 Details on the primers designed for the RT-PCR validation performed on mimicry candidate genes

Gene	Primer sequence (5'-3') Forward/Reverse	Amplicon length	Tm
WNT04	GTCATCAGCATTTCGACC / TCTTGCAGATACGGGCAA	219	58
Zn transporter	GACATCCGAAGTCTGCAAC / CCTCGTAGCCACCATTACCC	179	58
Lysyl oxidase	CGGAGACCAAGGAATAAG / TTTGCGTACGAAACTTCAGG	153	58
Palmitoyltransferase	CTATCACATGCCTGAAGACG / ATTTGCTGGAAGTGGGCTAA	156	56
EF1-α	CATCGATGACTGTGACGTTG / GAAGCCCAAGAGGTAAGTTT	175	58

Figures

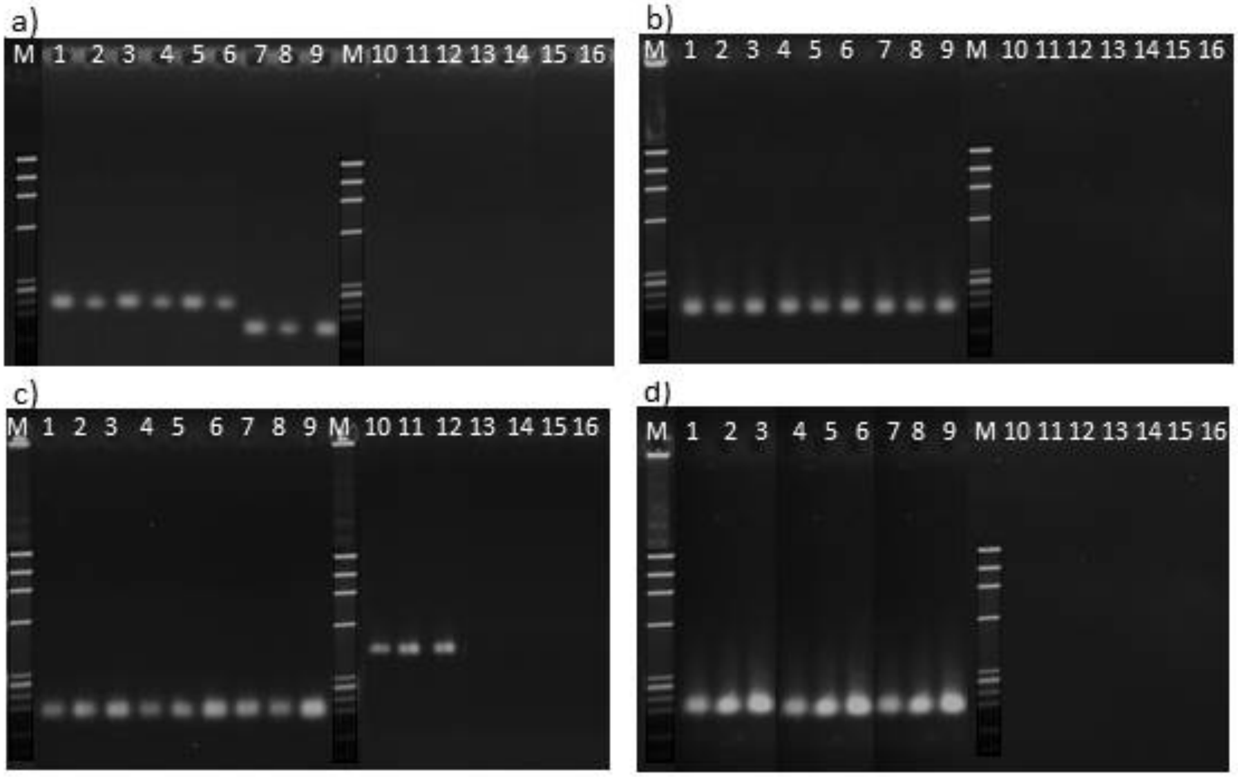

Figure S1 PCR-based validation of the four candidate mimicry genes in *Schistocephalus solidus*. In a) *membrane zinc transporter,* b) *Wnt04,* c) *palmitoyltransferase* and d) *lysyl oxidase homolog 2B*. Well M: molecular marker Φx174 DNA *Hae*III digest. Wells 1-3: coradicia genomic DNA samples (three pools from three different mating pairs). Wells 4-6: adult parasite genomic DNA. Wells 7-9: adult parasite cDNA. Wells 10-12: adult threespine stickleback genomic DNA. Wells 13-15: adult threespine stickleback cDNA. Well 16: negative control. The molecular marker produces band at 1353 bp, 1078 bp, 872 bp, 603 bp, 310 bp, 271-281 pb, 234 bp, 194 bp, 118 bp and 72 bp.
